# Moderators of long-term treatment outcome when comparing two group interventions for adolescents with ADHD: who benefits more from DBT-based skills training?

**DOI:** 10.1186/s12888-022-04435-8

**Published:** 2022-12-06

**Authors:** Jenny Meyer, Vendela Zetterqvist, Maria Unenge Hallerbäck, Mia Ramklint, Johan Isaksson

**Affiliations:** 1 Department of Medical Sciences, Child and Adolescent Psychiatry Unit, Uppsala University, Uppsala, Sweden; 2 Center of Neurodevelopmental Disorders (KIND), Centre for Psychiatry Research, Department of Women’s and Children’s Health, Karolinska Institutet, Stockholm, Sweden; 3 Department of Clinical Neuroscience, Karolinska Institute, Stockholm, Sweden; 4 School of Medical Sciences, Faculty of Medicine and Health, Örebro University, Örebro, Sweden; 5 Public Health Sciences, Karlstad University, Karlstad, Sweden

**Keywords:** ADHD, Adolescents, Cognitive behavioral therapy, Dialectical behavioral therapy, Psychoeducation, Moderation

## Abstract

**Background:**

Psychosocial interventions for adolescents with attention-deficit/hyperactivity disorder (ADHD), targeting emotional dysregulation and impulsive behaviors, have been requested, but the heterogeneity within this group makes it unlikely that there is one treatment that fits all. The aim of this study was to identify which adolescents with ADHD might have an effect from a structured skills training group (SSTG) based on dialectical behavioral therapy, by exploring pre-treatment characteristics as potential moderators of long-term treatment outcome.

**Methods:**

This study was based on follow-up data from a randomized controlled trial comparing the SSTG (*n* = 71) to a psychoeducational control intervention (*n* = 57) for adolescents with ADHD (15–18 years old). Clinical characteristics (sex, age, medication status, ADHD presentation, severity of ADHD symptom, psychiatric comorbidity, impairment of emotional dysregulation and functional impairment) were explored as potential moderators of pre-treatment to follow-up change in ADHD symptoms and functional impairment. Moderation analyses were performed using the PROCESS macro for SPSS.

**Results:**

Three moderators (severity of hyperactivity/impulsivity, conduct problems and impairment of emotional dysregulation) were identified in regard to the outcome self-rated change in ADHD symptoms. Participants with elevated pre-scores on these variables had a better effect of the SSTG than of the psychoeducational control intervention. No moderators were found in regard to the parental-rated outcomes.

**Conclusions:**

The SSTG seems to be beneficial for adolescents with ADHD who perceive pronounced symptoms of hyperactivity/impulsivity, conduct problems and emotional dysregulation. Our findings need to be confirmed in future trials evaluating dialectical behavioral therapy-based skills training for adolescents with ADHD, where these moderators could be used as criteria for inclusion or stratification.

**Trial registration:**

https://doi.org/10.1186/ISRCTN17366720, retrospectively registered.

**Supplementary Information:**

The online version contains supplementary material available at 10.1186/s12888-022-04435-8.

## Background

Attention-deficit/hyperactivity disorder (ADHD) is a neurodevelopmental condition characterized by symptoms of inattention, hyperactivity and impulsivity [1]. Adolescents with ADHD are a heterogenous group, with the presentation of ADHD symptoms, as well as associated difficulties and degree of impairment, varying across individuals [2–5]. About two-thirds of adolescents with ADHD have at least one additional psychiatric diagnosis, such as oppositional defiant disorder (ODD), conduct disorder (CD), anxiety disorders, depressive disorders, autism spectrum disorder or Tourette’s syndrome [6–8]. Many adolescents with ADHD display high emotional reactivity and difficulties with emotional regulation [9, 10]. Although functional impairment in daily activities is a diagnostic criterion of ADHD [1], the degree and type of impairment vary across individuals and settings [4, 5]. For example, while some adolescents with ADHD are affected by impairments mainly in school [11], others may experience more impairments in social activities [12, 13].

The heterogeneity among adolescents with ADHD makes it unlikely that there is one treatment that fits all [3]. While pharmacological treatment has been shown to be effective in reducing the core symptoms of ADHD [14], not all patients respond well to medication [15, 16]. Moreover, the adherence to ADHD medication is particularly low among adolescents [16, 17]. For these reasons, psychosocial interventions based on cognitive behavioral therapy (CBT) have been suggested as a possible complement for adolescents with ADHD [18]. However, psychosocial treatments for this group have shown inconsistent effects on both ADHD symptoms and functional impairment, and the evidence of long-term effects is limited [19, 20]. Accordingly, a need for more research on long-term outcomes and exploration of potential treatment moderators has been emphasized [19, 21].

Randomized controlled trials (RCTs) are regarded as the gold standard method for evaluating the effectiveness of interventions [22]. While the main analyses used in RCTs investigate the overall effectiveness of a treatment, moderator analyses can be used to identify under what circumstances and for whom a treatment is effective, and for which patients other interventions might be more appropriate [22–24]. In the context of an RCT, a moderating effect is present when the treatment effect (i.e., the difference in outcome between the treatment conditions) differs depending on the baseline value of an additional variable (i.e., a moderator). Overgeneralization of both positive and negative findings in an RCT is a problem that can be minimized by performing moderator analyses [22]. In our recently conducted RCT, the effectiveness of a structured skills training group (SSTG) based on traditional CBT and dialectical behavioral therapy (DBT) was evaluated for adolescents with ADHD [25]. Both the SSTG and the psychoeducational control intervention were associated with reduction in symptoms and impairment, but no significant group differences were found. The study was conducted within a clinical context and included a heterogenous study population, where the symptomatology and degree of functional impairment varied across participants [25]. To identify whether specific subgroup(s) responded differently to these interventions, further investigation into potential moderators of treatment outcome is needed.

Moderators and predictors of treatment outcome of psychosocial interventions for adolescents with ADHD have been investigated only scarcely [3, 26–28]. Previous studies on children and adolescents have indicated that neither sex [3, 26, 28–30], age [3, 26, 30] nor medication status [3, 30] seems to have a decisive impact on treatment outcomes such as ADHD symptoms, psychiatric comorbidity or functional impairment. However, sex has been found to influence changes in ADHD symptoms of group-based psychoeducation and mindfulness training for adults with ADHD [31]. While females had a better effect than males from both psychoeducation and mindfulness training (sex as an unspecific predictor), males had a better effect from mindfulness training than from psychoeducation (sex as a moderator). As regards the context of age, the SSTG is a downward translation of a manual initially developed for adults [32, 33] and does not include any parent or teacher involvement. Since the treatment partly relies on the adolescents’ own ability of transferring the practiced skills into daily life, older adolescents, who might be more capable of self-management, could potentially have benefitted more from the SSTG.

Regarding type and severity of ADHD symptoms and psychiatric comorbidity, previous studies have revealed mixed findings. In one study evaluating the effect of a sleep intervention for children with ADHD, individuals with high severity of ADHD symptoms had a larger reduction in ADHD symptoms after treatment [30]. In the Multimodal treatment study of children with ADHD, remission in symptoms of ADHD was less common for children with high ADHD symptom burden at baseline [34]. This finding was only observed for children who received the interventions that included medication management, and not for those who were assigned to the behavioral intervention or community care [34]. In addition, neither severity of ADHD symptoms [3, 26] nor ADHD presentation [3, 26, 28] has been found to influence the effect of CBT for adolescents with ADHD. Previous studies have shown that comorbid anxiety can have a positive impact on treatment outcome (e.g., symptoms of ADHD and anxiety) of psychosocial treatments for youths with ADHD [3, 28, 29, 34, 35]. In contrast, patients with comorbid disruptive behaviors have shown poorer response to psychosocial interventions [27, 28]. For children with several comorbidities (anxiety and ODD/CD), a combination of medication and behavioral treatment seems to be most beneficial [34].

Overall, these findings indicate that degree of ADHD symptoms, symptoms of psychiatric comorbidity and sex can have an impact on treatment outcome for patients with ADHD. However, the response patterns across subgroups may vary in different study populations and need to be considered in relation to the specific interventions and outcomes used in each study. In our RCT, both interventions contained psychoeducation and strategies for planning and organization [25]. In addition, the SSTG included DBT-based skills training (e.g., mindfulness, behavior analysis, acceptance and social skills) and was thus a longer, more resource-intensive intervention [32, 33]. Since the SSTG specifically targets emotional dysregulation, impulsive behavior and interpersonal problems, the treatment might be most effective for patients who struggle with those aspects.

Due to the heterogeneity among adolescents with ADHD, exploration of potential treatment moderators is warranted [3, 19, 21]. In addition, more studies on the long-term outcomes of psychological interventions for this group have been requested [19]. To the best of our knowledge, no previous studies have explored moderators in relation to DBT-based treatments for adolescents with ADHD. Thus, the aim of this study was to identify which adolescents with ADHD might have a more beneficial effect of the DBT-based SSTG as compared with the psychoeducational control intervention, by exploring pre-treatment characteristics as potential moderators of long-term treatment outcome. More specifically: Do any of the pre-treatment characteristics sex, age, medication status, ADHD presentation, severity of ADHD symptoms, psychiatric comorbidity, impairment of emotional dysregulation and degree of functional impairment moderate the treatment outcome of change in ADHD symptoms and functional impairment?

## Methods

### Participants and procedure

This was an explorative study where the study sample stemmed from a multi-center RCT conducted at seven child and adolescent psychiatric (CAP) outpatient units in Sweden [25]. The procedure has been described in detail in previous papers [25, 36]. In brief, adolescents aged between 15 and 18 years, with a clinical diagnosis of ADHD, were invited to participate by their regular outpatient contact. Those interested attended an initial meeting at their local CAP unit for assessment of study eligibility and to receive more information about the study. The assessment of eligibility was conducted by clinical psychologists who interviewed the adolescents and their parents and retrieved the clinical ADHD diagnosis from the adolescents’ medical records. Exclusion criteria were severe depression, suicidality, psychosis, or bipolar disorder without stable medication, mental retardation, organic brain injury, autism spectrum disorder or current substance abuse. Pharmacological treatment for ADHD was allowed, but the families were informed to keep the medication stable during the treatment period. In addition, the participants were requested not to take part in any other psychosocial treatment during the study period. If an adolescent was eligible for the study and wanted to participate, written informed consent was obtained from them and their parents. The eligible participants (*n* = 184) were thereafter randomly assigned to one of the two treatment conditions, at a 1:1 ratio, using a computer-generated allocation sequence (https://www.randomizer.org), with separate sequence lists for each treatment center. The principal investigator (JI), who was blinded to the participants, performed the treatment allocation.

Ratings were completed by the adolescents and their parents before treatment (*n* = 164), two weeks post-treatment (*n* = 132) and at a follow-up six months post-treatment (*n* = 128). To minimize the number of analyses and to focus on the long-term outcomes, follow-up was selected as the primary endpoint in this study. Accordingly, the moderator analyses were based on data from the 128 participants who completed the follow-up measurements (*n* = 118 for self-ratings and *n* = 125 for parental ratings). Participants who completed the pre-treatment measures but none of the follow-up measures were regarded as internal drop-outs and were included only in the attrition analysis (*n* = 36). Randomized participants who did not complete the pre-treatment measures (*n* = 20) were defined as external drop-outs and were not included in the attrition analysis due to the lack of data. Reasons for external drop-out has been reported elsewhere [25].

### Interventions

Both the SSTG and the psychoeducational control intervention were delivered in a group format and included psychoeducation about ADHD and closely related difficulties (e.g., with planning, organizing, structuring daily routines and stress). Both challenges and strengths of ADHD were discussed. PowerPoint presentations were used to visualize the contents, and group discussions and homework assignments were included in both interventions.

### SSTG

The treatment is an age-adapted version of a manualized DBT-based group program originally developed for adults with ADHD [32, 33]. Specific age adaptions have been described elsewhere [36]. The SSTG consists of 14 weekly two-hour sessions, where each session has a specific theme related to ADHD and associated difficulties (e.g., psychiatric comorbidity, emotional dysregulation and relational problems). The SSTG includes psychoeducation, group discussions and continuous practicing of skills that stem from DBT, such as mindfulness, acceptance, behavioral chain analysis and social skills. In the RCT, each group was led by two therapists, who were clinicians working at the CAP units, of whom at least one was a psychologist and one was trained in DBT. A detailed description of the SSTG can be found in previous papers [25, 36].

### Control intervention

The control intervention is a manual-based psychoeducational group program named SKILLS, which was developed by members of the research team. The intervention consists of three two-hour sessions, where the main focus is psychoeducation about ADHD and closely related difficulties. The participants also receive a book describing tools to facilitate schoolwork. The control intervention does not contain any DBT-related components. Each group was led by two therapists, who were clinicians working at the CAP units. The psychoeducational control intervention has been described in greater detail in previous papers [25, 36].

### Measures

#### Potential moderators

The following pre-treatment characteristics were explored as potential moderators of treatment outcome.

Sex and age were derived from the participants’ personal identity numbers. Both sex and age were treated as dichotomous variables, where sex was categorized as male or female, and age was dichotomized into a younger (15–16 years) and an older (17–18 years) group, with each participant’s age rounded to the nearest full year.

The use of ADHD medication was reported by the parents and this variable was dichotomized as either no medication or use of medication.

Current ADHD presentation was assessed pre-randomization by clinical psychologists who interviewed the participants and their parents, using the Mini International Neuropsychiatric Interview for Children and Adolescents (MINI-KID) [37]. Current ADHD presentation was based on the number of prevalent symptoms in the preceding six months, and assessed in accordance with the fifth edition of the Diagnostic and Statistical Manual of Mental Disorders [1]. The participants were categorized into one of the three ADHD presentations: inattentive, hyperactive/impulsive or combined presentation. Participants who did not have enough symptoms for any of the three main presentations were categorized as unspecified ADHD. Since only a few adolescents (*n* = 3) were categorized into the hyperactive/impulsive presentation, this group was merged with the combined presentation, as both groups included patients with hyperactivity and impulsivity. ADHD presentation was treated as a categorical variable in the analyses.

Severity of ADHD symptoms was measured with self-ratings and parental ratings on the subscales of inattention and hyperactivity/impulsivity from the Adult ADHD self-report scale for adolescents (ASRS-A) [38, 39]. The questionnaire contains nine items for each respective subscale, corresponding to the diagnostic symptoms of ADHD. The occurrence of each symptom is measured on a 5-point scale from 0 (never) to 4 (very often). Severities of ADHD symptoms were treated as continuous variables, with higher scores indicating higher severity of symptoms (min = 0, max = 36 for each subscale).

Symptoms of depression and anxiety were assessed using self-ratings on the respective subscales of the Hospital Anxiety and Depression Scale [40, 41]. Each subscale consists of seven statements measured on a 4-point scale, ranging from 0 to 3, where higher scores indicate greater occurrence of symptoms (min = 0, max = 21 for each subscale). Symptoms of depression and anxiety were treated as continuous variables in this study.

Conduct problems were assessed using self-ratings and parental ratings on the subscale of conduct problems within the Strength and difficulties questionnaire (SDQ) [42–44]. This subscale consists of five statements (e.g., often fights/quarrels with other children or bullies them), where each item is measured on a 3-point scale ranging from 0 to 2, with higher scores indicating greater occurrence of disruptive behaviors (min = 0, max = 10). Conduct problems were treated as a continuous variable.

Impairment of emotional dysregulation was assessed using the following question from the questionnaire Impact of ADHD Symptoms, constructed by the research team [25]: “In the last week, how much has your wellbeing been affected by difficulties with controlling your emotions?” The item was answered on an 11-point scale ranging from 0 to 10, where 0 = not at all, 2 = a little, 5 = quite a lot, 8 = a lot and 10 = very much. A cut-off score of ≥ 5 (i.e., quite a lot to very much) was used to identify participants who were affected by difficulties with emotional regulation. Impairment of emotional dysregulation was treated as a dichotomous variable.

Functional impairment in daily activities was assessed using self-ratings and parental ratings on the Swedish version of the Child Sheehan Disability Scale (CSDS) [45]. The self-rating scale contains three items that assess the impact of the adolescents’ troubles and feelings in school, social activities, and at home. Each item is measured on an 11-point scale from 0 (not at all) to 10 (very much) and the total score for the self-rating scale ranges between 0 and 30 points. The parental rating scale contains five items which assess the impact of the adolescent’s troubles and feelings in school, social activities, home, as well as on parents’ work and social activities (min = 0, max = 50). Functional impairment was treated as a continuous variable.

#### Treatment outcomes

The treatment outcomes were defined as the change in ADHD symptoms and functional impairment between the pre-treatment assessment and the follow-up assessment. Change in ADHD symptoms was assessed using self-ratings and parental ratings on the full scale of ASRS-A [38, 39]. Change in functional impairment was assessed using self-ratings and parental ratings on the full scale of CSDS [45].

#### Other measures

Use of ADHD medication during the study was categorized as: stable medication (i.e., continued with ADHD medication throughout the study period), major change of medication (i.e., either stopped or started using ADHD medication) or no medication (i.e., did not use any ADHD medication during the study).

Attendance at the group sessions was registered by the group leaders for each treatment condition (max 14 sessions in the SSTG and max 3 sessions in the control condition).

### Analyses

The original power calculation determining the sample size for the RCT was based on the main analyses and has been described in previous papers [25, 36]. A supplementary power calculation was performed for the moderator analyses, using G^*^ power, version 3.1. Specifically, to obtain a moderate effect size (f^2^ = 0.15), with a power of 80%, *a* = 0.05, a sample size of at least 77 participants was needed. All analyses were conducted with IBM SPSS Statistics, version 27.0. Baseline differences between the two treatment groups were examined for all potential moderators, for changes in medication and attendance at sessions, using t-tests for continuous variables and chi-squared tests for the dichotomous and categorical variables. The same statistics were used for the attrition analysis, where the study sample was compared to the internal drop-outs. Potential moderators were explored using the PROCESS macro for SPSS [46]. The changes (follow-up score minus pre-treatment score) in ADHD symptoms and functional impairment were included as dependent variables. The dummy variable representing the treatment condition (0 = control group and 1 = SSTG), the potential moderator variable and the interaction between treatment condition and the potential moderator, were all included as independent variables. A moderating effect was defined as a significant interaction between the treatment condition and the moderator on the outcome, i.e., when the two interventions led to significantly different effects in relation to different levels on the moderator. To preserve power, each potential moderator was introduced to the equation separately. As a sensitivity analysis, the significant moderators were analyzed again while adjusting for the other significant moderators entered as covariates in the model. No multicollinearity was found (the variance inflation factors were below 2.0). The Johnson-Neyman technique was used for the continuous moderators to identify the point(s) along the moderator where the relationship between treatment condition and outcome transitioned from being nonsignificant to being statistically significant [47]. Figures were used to illustrate the significant interactions and simple linear regression analyses were performed for each group separately to clarify the predictive value for the significant moderators on the treatment outcome(s). All reported beta coefficients are unstandardized. Given the explorative nature of this study, no adjustment for multiple testing was made. All the reported results were considered significant at the 5% level.

## Results

### Sample characteristics, attrition and adherence

Sample characteristics before treatment exposure are described in Table 1. No differences between the two treatment groups were found in any of the variables at baseline. However, a significant group difference was identified in regard to proportion of attendance at sessions (*t* = 4.10, *p* < 0.001). Specifically, the SSTG had an average of 70.0% attendance (mean = 9.8 sessions, *SD* = 3.69) and the control group had an average of 87.7% attendance (mean = 2.6 sessions, *SD* = 0.65). Most of the participants in both groups had pharmacological treatment for ADHD when entering the study (71.8% in the SSTG and 82.5% in the control group), and a majority of these participants continued with ADHD medication throughout the study (86.3% in the SSTG and 82.6% in the control group). One fifth of the participants underwent a major change in ADHD medication (i.e., either stopped or started with medication) during the study (21.1% in the SSTG and 17.9% in the control group), while some participants had no ADHD medication (16.9% in the SSTG and 12.5% in the control group). No significant group differences were found in regard to patterns/changes in medication. The attrition analysis identified one significant group difference for parental-rated inattention (*t* = 2.13, *p* = 0.035), where the internal drop-outs had a higher score (mean = 27.70) than those who completed the follow-up assessment (mean = 25.45). No other significant differences were found in the attrition analysis.

**Table 1:** Sample characteristics before treatment exposure

**Characteristics**	**SSTG *n* = 71**	**Control group *n* = 57**^a^
**Sex, n (%)**		
Females	44 (62.0)	36 (63.2)
**Age (years)**		
Mean (SD)	16.52 (0.91)	16.68 (0.95)
Younger (15–16), n (%)	29 (40.8)	22 (38.6)
Older (17–18), n (%)	42 (59.2)	35 (61.4)
**ADHD medication, n (%)**		
Use of medication	51 (71.8)	47 (82.5)
**ADHD presentation (MINI-KID), n (%)**		
Combined	27 (38.0)	28 (49.1)
Inattentive	27 (38.0)	19 (33.3)
Hyperactive/impulsive	2 (2.8)	1 (1.8)
Unspecified^b^	15 (21.1)	9 (15.8)
**ADHD symptoms, mean (SD)**		
Self-rated hyperactivity/impulsivity	17.94 (7.42)	19.46 (8.61)
Self-rated inattention	22.30 (6.96)	23.59 (6.52)
Parental rated hyperactivity/impulsivity	18.11 (6.73)	16.84 (8.20)
Parental rated inattention	26.30 (5.17)	24.40 (5.74)
**Symptoms of psychiatric comorbidity, mean (SD)**		
Self-rated anxiety	8.99 (4.76)	9.49 (4.14)
Self-rated symptoms of depression	6.44 (3.82)	5.96 (3.46)
Self-rated conduct problems	2.80 (1.65)	3.00 (2.05)
Parental rated conduct problems	2.87 (1.96)	2.51 (1.96)
**Impairment of emotional dysregulation, n (%)**^c^		
Much impairment	38 (53.5)	28 (50.0)
Little impairment	33 (46.5)	28 (50.0)
**Functional impairment, mean (SD)**		
Self-rated impairment	14.13 (7.39)	14.34 (7.10)
Parental rated impairment	25.94 (10.10)	23.42 (11.23)

*ADHD* Attention-deficit/hyperactivity disorder, *MINI-KID* Mini International Neuropsychiatric Interview for Children and Adolescents, *SD* standard deviation, *SSTG* structured skills training group

^a^For the self-ratings, *n* = 56

^b^Unspecified ADHD includes participants who did not fulfill the criteria for any of the main presentations in the MINI-KID interview

^c^Assessed using a question from the questionnaire Impact of ADHD Symptoms, constructed by the research team, where a score ≥ 5 was categorized as much impairment and a score ≤ 4 was categorized as little impairment

### Moderators

**Fig. 1:**
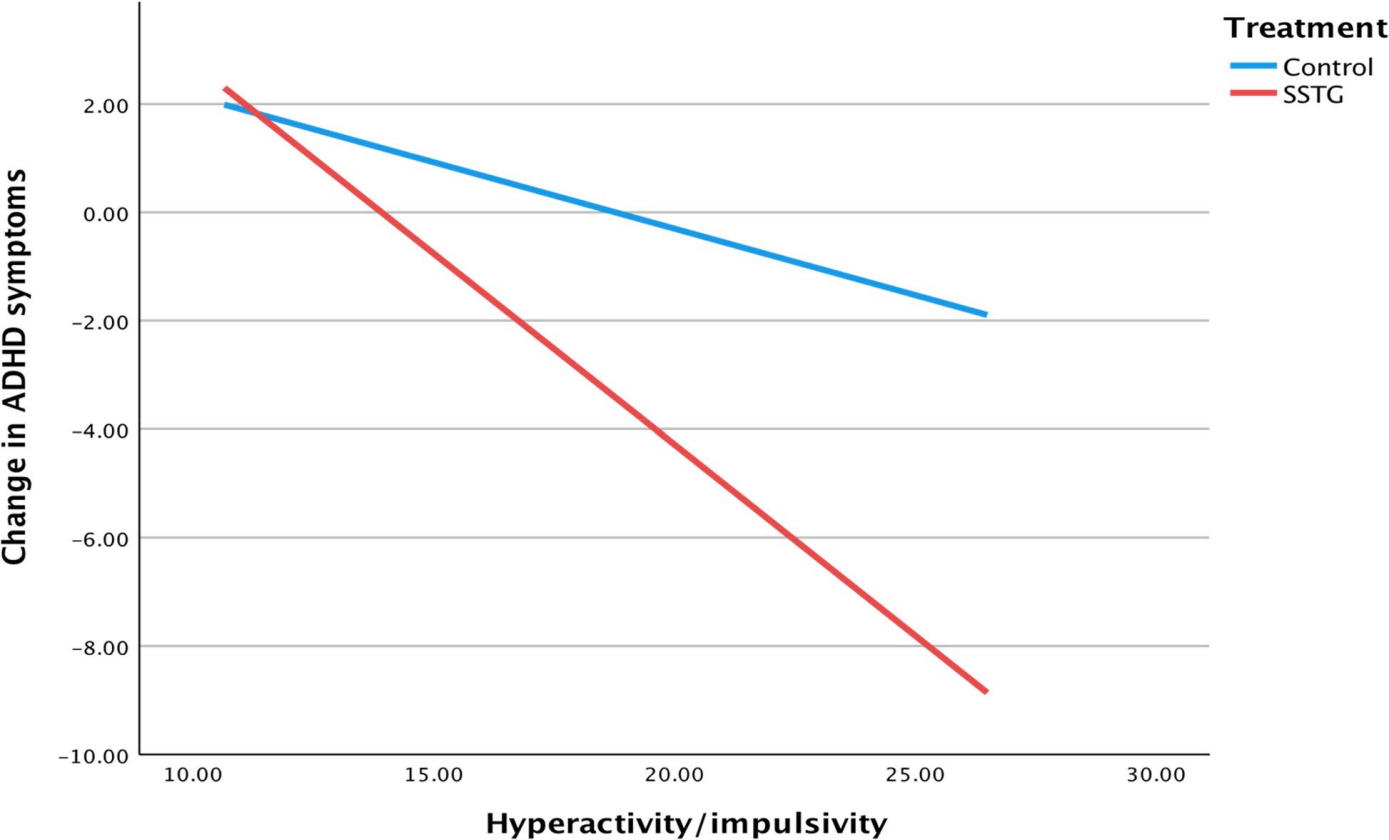
Interaction between treatment condition and severity of hyperactivity/impulsivity on self-rated change of ADHD symptoms

**Fig. 2:**
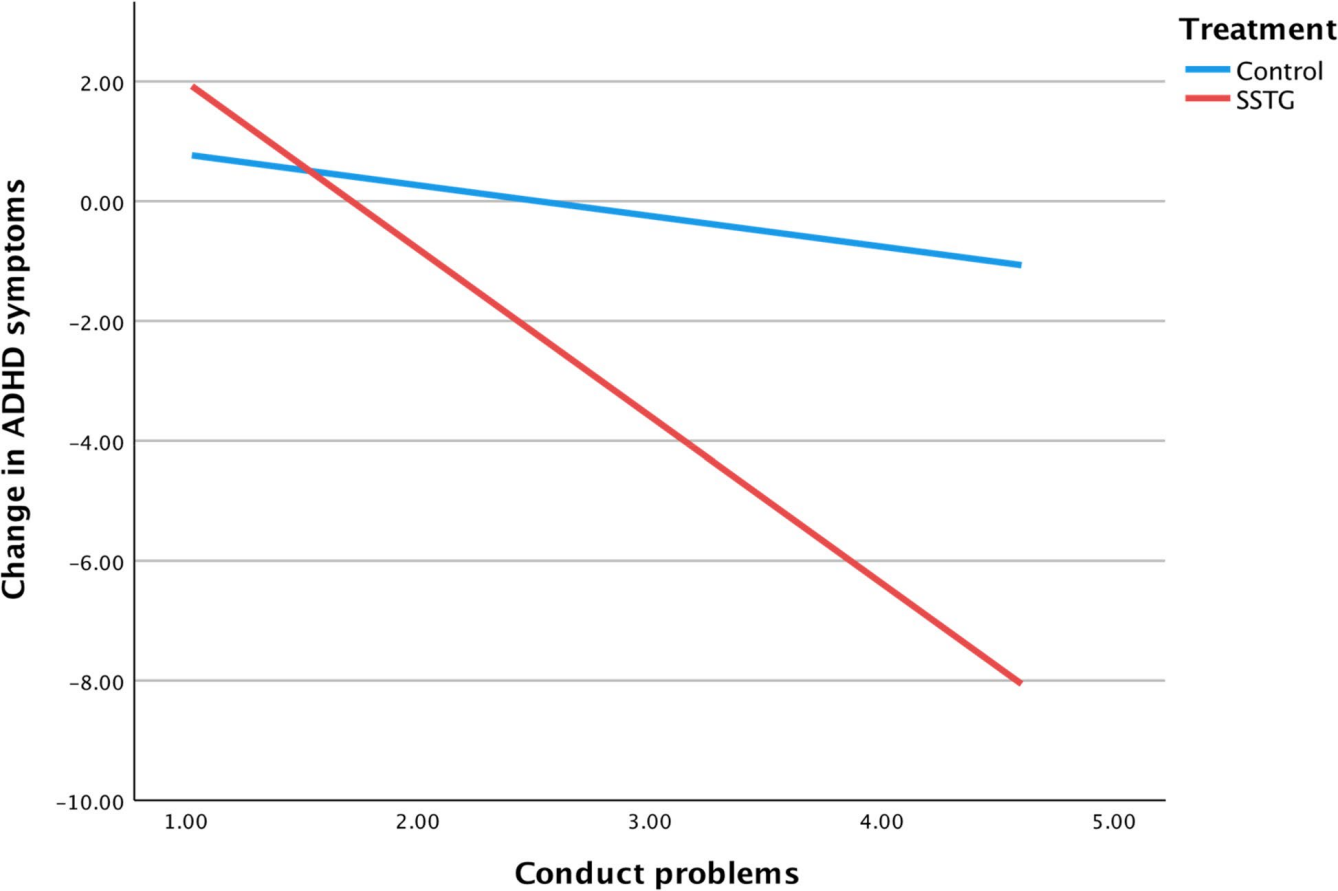
Interaction between treatment condition and conduct problems on self-rated change of ADHD symptoms

**Fig. 3:**
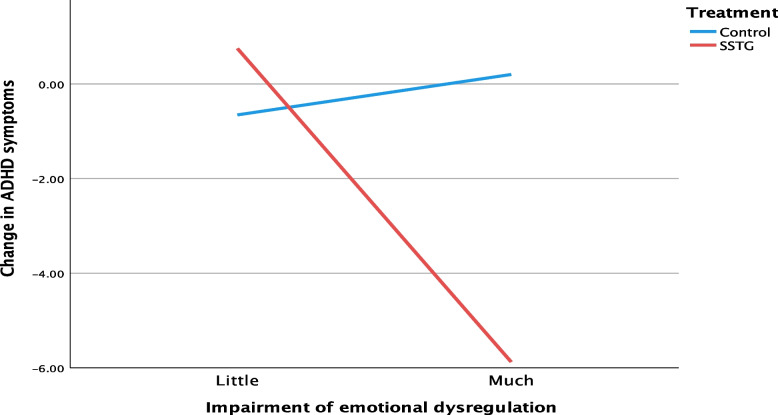
Interaction between treatment condition and impairment of emotional dysregulation on self-rated change of ADHD symptoms

As illustrated in Figs. 1, 2 and 3, three significant interactions were found. Specifically, self-rated severity of hyperactivity/impulsivity, conduct problems and impairment of emotional dysregulation were identified as moderators in regard to the outcome of self-rated change in ADHD symptoms.

For pre-treatment symptoms of hyperactivity/impulsivity, the interaction effect with treatment condition on changes in ADHD symptoms (*b* = -0.46, *p* = 0.031, 95% CI = -0.88; -0.04) showed that the SSTG was more effective than the control intervention for participants with higher severity of hyperactivity/impulsivity. A significant group difference was present from ≥ 19 points on the ASRS-A hyperactivity/impulsivity subscale. The sensitivity analysis confirmed the interaction (*b* = -0.47, *p* = 0.029, 95% CI = -0.89; -0.05). A one-point increase on the prescore of hyperactivity/impulsivity was associated with a 0.71 (95% CI = -1.02; -0.39, *R*^2^ = 0.23) point decrease in ADHD symptoms for the SSTG, while the corresponding estimate for the control group was *b* = -0.25 (95% CI = -0.51; 0.21, *R*^2^ = 0.07).

Moreover, an interaction with treatment condition and conduct problems on changes in ADHD symptoms was found in favor of the SSTG (*b* = -2.28, *p* = 0.021, 95% CI = -4.21; -0.34). A significant group difference was identified from the value of 3.2 of the SDQ subscale; this continued to be significant as the value of the moderator increased. Accordingly, for participants who had elevated symptoms of conduct problems, the SSTG was more effective than the control intervention. The interaction remained significant in the sensitivity analysis (*b* = -2.12, *p* = 0.028, 95% CI = -4.01; -0.23). A one-point increase in the subscale of conduct problems was associated with a 2.79 (95% CI = -4.42; -1.16, *R*^2^ = 0.15) point decrease on ADHD symptoms for the SSTG. Conduct problems was not a significant predictor of change in ADHD symptoms for the control group (*b* = -0.51, 95% CI = -1.61; 0.58, *R*^2^ = 0.02).

The interaction between treatment condition and impairment of emotional dysregulation on changes in ADHD symptoms (*b* = -7.48, *p* = 0.035, 95% CI = -14.44; -0.53) demonstrated that participants who reported much impairment of emotional dysregulation (i.e., quite a lot to very much) had a greater effect from the SSTG than from the control intervention. This finding was confirmed in the sensitivity analysis (*b* = -9.27, *p* = 0.006, 95% CI = -15.83; -2.70). The participants in the SSTG who reported much impairment of emotional dysregulation had a 6.63 larger decrease in ADHD symptoms than those who reported little impairment (*b* = -6.63, 95% CI = -11.65; -1.61, *R*^2^ = 0.10). In the control group, a nonsignificant increase of ADHD symptoms was observed for the participants who reported much impairment of emotional dysregulation (*b* = 0.85, 95% CI = -3.75; 5.47, *R*^*2*^ = 0.00) when compared with those with little impairment.

Neither sex, age, medication status, ADHD presentation, severity of inattention, anxiety, depression nor functional impairment moderated the change in self-rated ADHD symptoms and no moderation effect was found regarding the change in self-rated functional impairment. Furthermore, none of the pre-treatment characteristics moderated the parental-rated outcomes. The results from all moderator analyses are presented in Additional file 1.

## Discussion

This is the first study exploring treatment moderators of a DBT-based structured skills training group for adolescents with ADHD. Three treatment moderators were identified in regard to the outcome self-rated change in ADHD symptoms. For participants who perceived pronounced symptoms of hyperactivity/impulsivity, conduct problems and/or impairment of emotional dysregulation, the SSTG was superior to the psychoeducational control intervention. In contrast, the results did not reveal any specific subgroup(s) where the SSTG outperformed the control group regarding the effect on functional impairment. In addition, no moderators were found in regard to the parental-rated outcomes.

DBT was originally developed for patients with suicidal behaviors and borderline personality disorder (BPD) [48]. The overlap in symptoms between ADHD and BPD (e.g., emotional dysregulation, impulsive behaviors) [49, 50] is often mentioned when suggesting DBT as a potential treatment method for patients with ADHD [51–54]. The three moderators identified in this study (hyperactivity/impulsivity, conduct problems and impairment of emotional dysregulation) correspond to this overlap, indicating that the patients who struggle with these symptoms are the ones who benefit most from the treatment. In contrast to our findings, previous studies have demonstrated a poorer response to psychosocial treatments for youths with disruptive behaviors [27, 28]. The continuous practicing of mindfulness and behavioral chain analysis in the SSTG could help adolescents become more aware of their behavioral patterns (i.e., triggers, non-adaptive behaviors, consequences) and create more room for self-regulation to enable the use of goal-oriented behaviors [55, 56]. In line with our findings, previous studies have indicated that DBT-based skills training could be helpful for patients with disruptive behaviors [57, 58]. In the current study, conduct problems were assessed using questionnaires and it is uncertain if similar results would have been found for patients with a clinical diagnosis of CD, ODD or disruptive mood dysregulation disorder. Although our results indicated that the SSTG could be a promising treatment for certain subgroups of adolescents with ADHD, these findings are derived from exploratory analyses and need to be confirmed in future trials where the identified moderators could be used as criteria for inclusion or stratification [22].

However, far from all patients with ADHD have pronounced symptoms of hyperactivity, impulsivity, disruptive behavior and emotional dysregulation [1, 9, 10]. For the participants who did not have these pronounced symptoms, the SSTG was not proved to be more effective than the psychoeducational control intervention. Some of the participants had rather stable ADHD symptoms at baseline, possibly due to medication, and there might have been limited room for further symptom reduction for these individuals. These patients might still benefit from meeting peers with ADHD and learning more about their diagnosis. However, for this purpose, a shorter psychoeducational group intervention might be enough. Moreover, there are likely subgroups where none of these interventions will be a satisfying treatment option. For example, severity of inattention was not a moderator in this study and the attrition analysis indicated that participants with high severity of inattention were at larger risk of drop-out. Although mindfulness training has been associated with improvement in symptoms of inattention, the evidence of its effectiveness remains limited [31, 55, 59]. Inattention is strongly related to academic impairment [60], meaning that patients who have severe attentional deficits probably need more extensive practicing to improve skills in planning and organization, for example.

No moderation effect was observed for the parental-rated outcomes, in contrast to the self-ratings. Hence, the parental ratings did not confirm the superiority of the SSTG for any subgroup, why the results based on the self-ratings need to be interpreted with some caution. In the main analysis of the RCT [25], the parental ratings indicated a larger decrease of ADHD symptoms as compared to the self-ratings, a finding that was seen in both treatment conditions [25]. Accordingly, the parents appeared to be more optimistic about change in symptoms regardless of which treatment group their adolescents were assigned to or what characteristics their adolescents had prior to the treatments. At the same time, we want to emphasize the clinical value of the adolescents’ own perspective, since they are the ones who participated in the treatments [20, 61]. In order to offer interventions that are perceived as relevant and effective by the adolescents themselves, self-rated moderators are likely to be of great importance.

The absence of moderators regarding the effect of functional impairment showed that even when pre-treatment characteristics were considered, the SSTG was not superior to the control group in reducing functional impairment. Hypothetically, to achieve improvement in daily adaptive functions at home or in school, more extensive practicing of skills that are needed in those specific contexts might be necessary [20, 62]. In addition, involvement of parents and teachers in the treatment could support the adolescents’ practicing and use of the skills in their daily life and could also facilitate the implementation of the environmental adaptions that are needed [63]. Neither sex, age, medication status nor ADHD presentation was identified as moderators, which is consistent with previous studies on adolescents with ADHD [3, 26]. In regard to age, the restriction of range (15–18 years) may have influenced the results. In contrast to earlier findings [3, 29, 34, 35], anxiety was not identified as a moderator in this study.

This study has some limitations that need to be considered. The pre-treatment measures were assessed after randomization, we included only participants who had completed the follow-up measurements and the study suffered from relatively large drop-out. Hence, there is a risk of selective drop-out, restricted power for moderator analyses and systematic differences between the groups. However, the comparative analyses of the two treatment conditions indicated no systematic group differences before treatment exposure. The attrition analysis did reveal one difference, indicating that patients with severe attentional deficits had more problems with completing follow-up measures. Apart from that, the study sample corresponded well to the original sample used in the RCT [25]. Since the results from this study are based on explorative analyses, the findings need to be replicated in future RCTs before any firm conclusion can be drawn [22].

Although clinically relevant characteristics were explored as potential moderators, several factors were not measured in this study and were therefore not included. Demographic factors such as socioeconomic status, degree of parent-teen conflict, parental stress and parental depression have previously been shown to have an impact on treatment outcome for youths with ADHD [29, 64] and these factors should be investigated in future trials. Moreover, in contrast to the other moderators which were measured with validated rating scales, impairment of emotional dysregulation was assessed with a single item from a questionnaire developed by the research team [25, 36]. The lack of a validated rating scale of emotional dysregulation, such as the Difficulties in Emotion Regulation Scale [65], should be regarded as a limitation which makes the conclusions more uncertain. Lastly, although self-ratings and parental ratings could be a valid method for assessing symptoms [61], the use of a more objective assessment, performed by a blinded clinician, would have been warranted.

This study also has some strengths. The relatively large sample size of the RCT enabled the exploration of potential treatment moderators. Due to the research gap on long-term effects in this area [19], the focus on long-term outcomes could also be regarded as a strength. In addition, this study was conducted within a clinical setting, which may have strengthened the ecological validity of the study findings.

## Conclusions

Because of the clinical heterogeneity among adolescents with ADHD, the exploration of treatment moderators is crucial to identify which patients benefit from a certain treatment [3, 19, 21]. This is the first study that explores moderators of the long-term outcomes from a DBT-based skills training group for adolescents with ADHD. In regard to the outcome of change in ADHD symptoms, our findings revealed that the SSTG seemed to be an effective treatment for adolescents with ADHD who perceive pronounced symptoms of impulsivity/hyperactivity, conduct problems and/or impairment of emotional dysregulation. The absence of moderators in regard to the outcome of functional impairment confirmed the overall findings from the RCT [25] and indicated that further adaptions of the SSTG are needed to create evident improvements of functioning in daily life. Though our findings give some preliminary indications of which patients might respond best to the SSTG, we primarily regard the results from this study as hypothesis-generating, to guide researchers in the best choice of inclusion criteria or stratification to maximize power in future trials.

## Supplementary Information

**Additional file 1: Table 1.** Interactions between treatment condition and potential moderators of self-rated change in ADHD symptoms (*n*=118). **Table 2.** Interactions between treatment condition and potential moderators of self-rated change in functional impairment (*n*=118). **Table 3.** Interactions between treatment condition and potential moderators of parent-rated change in ADHD symptoms (*n*=125). **Table 4.** Interactions between treatment condition and potential moderators of parent-rated change in functional impairment (*n*=125).

## Data Availability

Consent for sharing individual data outside the research team was not obtained. However, reasonable requests for patient-level data can be made to the corresponding author (JI) and will be considered after discussion with the ethical review board. Relevant data are included in the manuscript.

## Abbreviations

ADHD: Attention-deficit/hyperactivity disorder
ASRS-A: Adult ADHD Self-Report Scale Adolescent version
BPD: Borderline personality disorder
CAP: Child and adolescent psychiatric
CBT: Cognitive behavioral therapy
CD: Conduct disorder
CSDS: Child Sheehan Disability Scale
DBT: Dialectical behavioral therapy
MINI-KID: Mini International Neuropsychiatric Interview for Children and Adolescents
ODD: Oppositional defiant disorder
RCT: Randomized controlled trial
SDQ: Strength and difficulties questionnaire
SPSS: Statistical Package for the Social Sciences
SSTG: Structured skills training group
