# Supplementary material for: Moderators of long-term treatment outcome when comparing two group interventions for adolescents with ADHD: who benefits more from DBT-based skills training?
Source: BMC Psychiatry. 2022 Dec 6;22:767. doi: 10.1186/s12888-022-04435-8 (PMC9724371; doi:10.1186/s12888-022-04435-8)
Supplement: Supplementary file 1 — Additional file 1: Table 1. Interactions between treatment condition and potential moderators of self-rated change in ADHD symptoms (n=118). Table 2. Interactions between treatment condition and potential moderators of self-rated change in functional impairment (n=118). Table 3. Interactions between treatment condition and potential moderators of parent-rated change in ADHD symptoms (n=125). Table 4. Interactions between treatment condition and potential moderators of parent-rated change in functional impairment (n=125). [file 12888_2022_4435_MOESM1_ESM.docx]

**Additional Table 1** Interactions between treatment condition and potential moderators of self-rated change in

ADHD symptoms (n=118)

| **Potential moderators** | ***b*** | **95%CI** | | ***p*** |
| --- | --- | --- | --- | --- |
|  |  | **lower** | **upper** |  |
| Sex | -2.16 | -9.69 | 5.38 | 0.572 |
| Age  ADHD medication | -0.00  5.61 | -7.38  -3.24 | 7.37  14.47 | 0.999  0.212 |
| ADHD presentation (MINI-KID)  Combined vs. inattention  Combined vs. unspecified ADHD  Inattention vs. unspecified ADHD | 0.15  -2.60  -2.75 | -7.67  -12.33  -12.80 | 7.97  7.13  7.29 | 0.969  0.597  0.588 |
| Severity of hyperactivity/impulsivity (ASRS-A)^a^ | -0.46 | -0.88 | -0.04 | **0.031** |
| Severity of inattention (ASRS-A) | -0.43 | -0.95 | 0.10 | 0.110 |
| Symptoms of anxiety (HADS) | -0.12 | -0.93 | 0.70 | 0.779 |
| Symptoms of depression (HADS) | -0.42 | -1.43 | 0.59 | 0.417 |
| Conduct problems (SDQ) | -2.28 | -4.21 | -0.34 | **0.021** |
| Impairment of emotional dysregulation^b^ | -7.48 | -14.44 | -0.53 | **0.035** |
| Functional impairment (CSDS) | -0.13 | -0.61 | 0.36 | 0.610 |

*Note*. The results were obtained from separate models. Beta coefficients are unstandardized. Significant interactions are printed in bold black.

*ADHD* attention-deficit/hyperactivity disorder*, ASRS-A* Adult ADHD self-report scale for adolescents, *CSDS* Child Sheehan

Disability Scale, *HADS* Hospital Anxiety and Depression Scale, *MINI-KID* Mini International Neuropsychiatric Interview for

Children and Adolescents, *SDQ* Strength and difficulties questionnaire.

^a^Due to missing data for one participant on the variable severity of hyperactivity/impulsivity, n = 117 for this analysis

^b^ Assessed using a question from the questionnaire Impact of ADHD symptoms, constructed by the research team, where a score ≥ 5 was categorized as much impairment and a score ≤ 4 was categorized as little impairment.

**Additional Table 2** Interactions between treatment condition and potential moderators of self-rated change in

functional impairment (n=118)

| **Potential moderators** | ***b*** | **95%CI** | | ***p*** |
| --- | --- | --- | --- | --- |
|  |  | **lower** | **upper** |  |
| Sex | -0.47 | -6.30 | 5.37 | 0.874 |
| Age  ADHD medication | 3.87  0.45 | -1.78  -6.45 | 9.51  7.36 | 0.177  0.897 |
| ADHD presentation (MINI-KID)  Combined vs. inattention  Combined vs. unspecified ADHD  Inattention vs. unspecified ADHD | -0.29  -2.10  -1.81 | -6.47  -9.80  -9.76 | 5.90  5.61  6.14 | 0.927  0.591  0.653 |
| Severity of hyperactivity/impulsivity (ASRS-A)^a^ | -0.18 | -0.53 | 0.16 | 0.299 |
| Severity of inattention (ASRS-A) | 0.06 | -0.36 | 0.47 | 0.791 |
| Symptoms of anxiety (HADS) | -0.18 | -0.45 | 0.81 | 0.569 |
| Symptoms of depression (HADS) | -0.50 | -1.27 | 0.27 | 0.199 |
| Conduct problems (SDQ) | -0.08 | -1.65 | 1.49 | 0.918 |
| Impairment of emotional dysregulation^b^ | -1.98 | -7.21 | 3.25 | 0.454 |
| Functional impairment (CSDS) | -0.09 | -0.41 | 0.23 | 0.586 |

*Note*. The results were obtained in separate models. Beta coefficients are unstandardized

*ADHD* attention-deficit/hyperactivity disorder*, ASRS-A* Adult ADHD self-report scale for adolescents, *CSDS* Child Sheehan

Disability Scale, *HADS* Hospital Anxiety and Depression Scale, *MINI-KID* Mini International Neuropsychiatric Interview for

Children and Adolescents, *SDQ* Strength and difficulties questionnaire.

^a^Due to missing data for one participant on the variable severity of hyperactivity/impulsivity, n = 117 for this analysis

^b^Assessed using a question from the questionnaire Impact of ADHD symptoms, constructed by the research team, where a score ≥ 5 was categorized as much impairment and a score ≤ 4 was categorized as little impairment.

**Additional Table 3** Interactions between treatment condition and potential moderators of parent-rated change in

ADHD symptoms (n=125)

| **Potential moderators** | ***b*** | **95% CI** | | ***p*** |
| --- | --- | --- | --- | --- |
|  |  | **lower** | **upper** |  |
| Sex | -1.53 | -7.99 | 4.93 | 0.640 |
| Age  ADHD medication | -2.21  1.49 | -8.58  -6.14 | 4.17  9.12 | 0.495  0.699 |
| ADHD presentation (MINI-KID)  Combined vs. inattention  Combined vs. unspecified ADHD  Inattention vs. unspecified ADHD | -0.87  -4.14  -3.27 | -7.90  -12.87  -12.29 | 6.16  4.59  5.75 | 0.806  0.350  0.475 |
| Severity of hyperactivity/impulsivity (ASRS-A) | -0.11 | -0.52 | 0.30 | 0.590 |
| Severity of inattention (ASRS-A) | -0.37 | -0.92 | 0.19 | 0.193 |
| Conduct problems (SDQ) | -0.36 | -1.95 | 1.22 | 0.651 |
| Functional impairment (CSDS) | 0.12 | -0.18 | 0.41 | 0.439 |

*Note*. The results were obtained in separate models. Beta coefficients are unstandardized

*ADHD* attention-deficit/hyperactivity disorder*, ASRS-A* Adult ADHD self-report scale for adolescents, *CSDS* Child Sheehan Disability

Scale, *MINI-KID* Mini International Neuropsychiatric Interview for Children and Adolescents, *SDQ* Strength and difficulties questionnaire.

**Additional Table 4** Interactions between treatment condition and potential moderators of parent-rated change in

functional impairment (n=125)

| **Potential moderators** | ***b*** | **95% CI** | | ***p*** |
| --- | --- | --- | --- | --- |
|  |  | **lower** | **upper** |  |
| Sex | 2.45 | -3.94 | 8.84 | 0.449 |
| Age  ADHD medication | -0.07  -0.40 | -6.31  -7.98 | 6.46  7.19 | 0.982  0.918 |
| ADHD presentation (MINI-KID)  Combined vs. inattention  Combined vs. unspecified ADHD  Inattention vs. unspecified ADHD | -3.18  -2.06  1.12 | -10.07  -10.61  -7.71 | 3.71  6.50  9.96 | 0.362  0.635  0.801 |
| Severity of hyperactivity/impulsivity (ASRS-A) | 0.03 | -0.39 | 0.45 | 0.884 |
| Severity of inattention (ASRS-A) | -0.21 | -0.77 | 0.35 | 0.462 |
| Conduct problems (SDQ) | 0.81 | -0.76 | 2.37 | 0.309 |
| Functional impairment (CSDS) | 0.19 | -0.09 | 0.47 | 0.191 |

*Note*. The results were obtained in separate models. Beta coefficients are unstandardized.

*ADHD* attention-deficit/hyperactivity disorder*, ASRS-A* Adult ADHD self-report scale for adolescents, *CSDS* Child Sheehan Disability Scale,

*MINI-KID* Mini International Neuropsychiatric Interview for Children and Adolescents, *SDQ* Strength and difficulties questionnaire.
